# Specific Physical Performances of Young Male Basketball Players in Palestine: An Assessment by Maturity Status

**DOI:** 10.3390/children12010064

**Published:** 2025-01-07

**Authors:** Layla Jawabreh, Mohamed Tounsi, Ghazi Racil, Johnny Padulo, Gian Mario Migliaccio, Luca Russo, Yassine Trabelsi

**Affiliations:** 1 Research Laboratory, Exercise Physiology and Physiopathology: From Integrated to Molecular “Biology, Medicine and Health” (LR19ES09), Faculty of Medicine of Sousse, University of Sousse, Sousse 4000, Tunisia; laylajawabreh@yahoo.com (L.J.); m.tounsi@hotmail.fr (M.T.); yassine.trabelsi@issep.uma.tn (Y.T.); 2 Research Unit (LR23JS01) “Sport Performance, Health & Society”, Higher Institute of Sport and Physical Education of Ksar Saîd, University of Manouba, Tunis 2010, Tunisia; ghazi.racil@issep.uma.tn; 3 Department of Biomedical Sciences for Health, Università degli Studi di Milano, 20133 Milan, Italy; 4 Department of Human Sciences and Promotion of the Quality of Life, San Raffaele Rome Open University, 00166 Rome, Italy; gianmario.migliaccio@uniroma5.it; 5 Research Unit Maxima Performa, Athlete Physiology, Psychology and Nutrition, 20126 Milano, Italy; 6 Department of Theoretical and Applied Sciences, eCampus University, 22060 Novedrate, Italy; luca.russo2@uniecampus.it

**Keywords:** growing, basketball, physical performance, stature

## Abstract

Objectives: There is a lack of studies that investigate the relationship between anthropometric profiles, biological maturity, and specific physical performances in young male basketball players. This study aimed to evaluate the development of anthropometric characteristics and physical performance across different age and maturity groups among male basketball players in Palestine, as well as to identify the anthropometric factors influencing physical performance within this population. Methods: A total of one-hundred-fifty male basketball players, aged 12 to 16, participated in this study. The players were categorized by age groups (U-12 to U-16) and divided into five maturational groups based on their maturity offset, ranging from −1.5 to 2.5 years relative to peak height velocity (PHV). The measurements included anthropometric variables and physical performance, such as sprinting performances, jumping ability, agility tests, and maximal aerobic capacity. Statistical analyses, including a full model and multiple regression analysis, were conducted to identify the anthropometric parameters that significantly influenced the performance variables. Results: Anthropometric development, particularly lower-limb length, significantly influenced vertical jump performance, while increases in body composition and fat mass negatively impacted sprinting and strength test outcomes. Our study confirms that maturity status plays a crucial role in determining physical performance among young Palestinian basketball players. Early-maturing players generally demonstrated greater strength and endurance, whereas late-maturing players excelled in agility and speed. The full model and the multiple equations were used to determine the determinants of physical performances according to anthropometric variables. Conclusions: The findings show that performance benchmarks based on age and maturational groups may contribute to a better understanding of training protocols and talent identification for young male basketball players in Palestine, as well as support the development of strategies for physical activity practice.

## 1. Introduction

Basketball is one of the most popular ball games around the world. It is a sport in which the ultimate success of the team depends largely on skills; physical, tactical, and physiological capacities; and mental preparation [1]. Therefore, a basketball player must possess morphological and physiological requirements, including high aerobic and anaerobic capabilities. Essential attributes include strength, agility, multidirectional movement, jumping ability, endurance, and sprinting performance [2].

For fitness coaches working with youth athletes, regular and ongoing anthropometric and physical assessments are essential. These performance metrics enable coaches to compare individual profiles and design training programs that align with athletes’ chronological age and developmental stage, taking into account each athlete’s unique biological age and maturity [3]. The variability of biological maturation, function, and physical abilities is a key factor contributing to players showing statistically significant differences in performance [4]. Various studies illuminate the complex interplay between maturity and physical performance through data and rigorous analysis, often utilizing advanced methods like bioimpedance analysis to evaluate body composition. Generally, males reach PHV at the age of 14 years, while females tend to reach accelerated growth at approximately 12 years of age [5]. Due to biological development, several studies have recommended the use of chronological age and peak height velocity (PHV) as biological indicators to monitor training programs and sessions [6]. This helps in controlling adaptation to training exposure, reducing injury risk, and consequently, enhancing the coaching effectiveness process [7,8]. Previous studies related to PHV physical abilities development according to age and maturity have been developed significantly in the youth category for males and females in various sports, focusing on explosive power, strength, and aerobic performance for individual and collective sports such as soccer [9,10], rugby [11], handball [12] and table tennis [13].

Research supports the notion that maturation significantly impacts physical performance metrics such as vertical jump height, sprinting velocity, and intermittent endurance capacity [10,14,15]. Gaining insight into the determinants that influence the development of physical characteristics during this critical developmental phase is vital for optimizing athletic performance and training strategies. Previous research has categorized athletes into three overarching maturational classifications: early, on time, and late maturing [16,17,18]. Consequently, further research is warranted to deepen understanding among coaches and practitioners, enabling them to adapt training protocols effectively in response to the dynamic changes induced by growth and maturation.

The relationship between anthropometric variables, maturation, and performance is shaped by biological processes that influence physical characteristics and functional capacities. Maturation impacts height, lean mass, and body composition, which directly affect performance metrics such as jumping ability, speed, and endurance [4,10]. These interconnected factors highlight the need for training programs that consider biological age and maturity to optimize development, reduce injury risk, and address potential selection biases [7,8].

In youth basketball, early-maturing athletes often exhibit significant advantages in anthropometric characteristics, such as height and lean mass, and physical capabilities, including jumping ability and sprint performance. These advantages frequently lead to selection biases, where early-maturing players are favored over their less-mature peers [19,20,21]. While studies on the impact of physical maturity on basketball performance are well-documented in regions such as Europe, North America, and North Africa [15,22,23], there is limited research addressing Middle Eastern athletes. Palestinian players are influenced by unique socio-cultural, environmental, and developmental factors that may alter growth and performance trajectories. These differences highlight the need for region-specific data to guide talent identification and training strategies. Relying solely on data from other regions risks misrepresenting the physical and performance characteristics of Palestinian players. This study aims to fill this gap by investigating performance metrics tailored to Palestinian youth basketball players, thereby contributing to a deeper understanding of maturity-associated performance in the Middle East.

To the best of our knowledge, no studies have been conducted to examine the effect of biological maturity, including PHV, on anthropometric variables and physical performances of young male basketball players in Palestine.

Hence, the aims of this study were (1) to assess the development of anthropometric characteristics and physical performance across different age and maturity groups among male basketball players in Palestine aged 12 to 16 and (2) to determine the anthropometric factors influencing physical performance in this population.

Based on previous findings in the literature, it is hypothesized that biological maturity, as indicated by PHV, will significantly influence anthropometric variables and physical performance among young male basketball players. Specifically, early-maturing players are expected to demonstrate greater strength and endurance, while late-maturing players are hypothesized to excel in agility and speed [22,24]. Additionally, analyzing physical performance trends across age groups is expected to provide valuable information for tailoring training protocols and identifying talent among Palestinian male basketball players aged 12 to 16 years.

## 2. Materials and Methods

### 2.1. Participants

One hundred and fifty male basketball players, ranging in age from 12 to 16 years, were divided into five age groups, with thirty players in each group, volunteering to participate in this study. To recruit a diverse sample of the target population, the participants were selected from 14 basketball clubs across different geographical regions in Palestine (north, center, and south), including two clubs each from Nablus, Ramallah, and Hebron. While this approach aimed to reflect the demographic and regional diversity of young basketball players in Palestine, we acknowledge that the sample may not be fully representative due to the absence of formal sample size calculations and logistical constraints. The experimental procedures were conducted during the months of July and August in 2022. The participants were categorized into age groups (U-12, U13, U-14, U-15, and U-16) based on their year of birth. All participants practiced basketball three times per week for an average of 70 min per session, a frequency and duration selected to ensure a baseline level of basketball-specific training that is consistent with youth athletic development guidelines and the logistical feasibility within the study context [25].

Assessments were conducted within three days for each region. On the first day was the anthropometric measurement, and on the second and third days were the physical measurements. The participants and their respective clubs were thoroughly informed about the nature and objectives of the study. The clubs, recognizing the potential benefits of the research findings for enhancing player development, consented to the participation of their young basketball players in the evaluation. Informed consent was obtained from both the players and their parents prior to the commencement of data collection, ensuring that all parties were fully aware of the study’s objectives and procedures. Additionally, formal approval for the study was secured from the Palestinian Ministry of Education, affirming adherence to ethical standards and regulatory requirements. The study protocol was approved by the Ethics Committee of the Faculty of Medicine “Ibn El Jazzar” of Sousse (Tunisia) (approval number: CEFMS 213/2023). It is noted that all measurements were conducted by a trained specialist researcher, namely a doctor with expertise in physical fitness assessment and anthropometric composition, to ensure consistency and accuracy.

### 2.2. Anthropometric Measurements

Height was measured in both standing and sitting positions using a portable stadiometer (seca 213 portable measuring rod, Chino, CA, USA). Arm length was measured from the acromion process to the radial styloid using an inextensible plastic tape. Wingspan was recorded as the distance from fingertip to fingertip with arms fully extended horizontally, while the length of the lower limb (LLL) was measured from the iliac crest to the lateral malleolus. Body weight, body mass index (BMI), body fat percentage, and fat-free mass were estimated through bioimpedance analysis (BIA) using a Human Body Element Analyzer (Detect Elements in the Body MSLCA05, Guangzhou, China). During the measurement, the individual maintained a neutral and relaxed posture, either standing upright for scale-based devices or lying flat for multi-frequency or segmental BIA devices. Bare skin was in direct contact with the electrodes, ensuring proper positioning per the device instructions. The pre-test guidelines included avoiding food for 2–4 h, limiting fluid intake, abstaining from strenuous physical activity for 12–24 h, and avoiding caffeine and alcohol for at least 24 h before testing.

#### Maturity Status

The determination of maturity status utilized the predictive equation for males developed by [26]. This approach was approved for predicting years from PHV, serving as an indicator of biological age through anthropometric variables according to predictive Equation (1).

Maturity Offset = −9.236 (0.0002708 × Length of lower limbs and Sitting height interaction) + (0.001663 × Age and Sitting height interaction) + (0.007216 × Length of lower limbs by Height ratio) + (0.02292 × Age and Weight interaction).(1)

According to a study by Rumpf, et al. [27], the protocol estimates the time before or after PHV from chronological age using anthropometric variables (height, sitting height, body mass index, and leg length) from the study of Mirwald, Baxter-Jones, Bailey and Beunen [26]. A chronological age of approximately 0.1 years was calculated by subtracting the date of birth from the date of testing. Negative values indicating time before PHV were added to chronological age and positive values indicating time after PHV were subtracted.

The maturity status and classification of groups were according to the study of [28]. The player was categorized into 1 of 6 maturity-offset groups (−2.5 YPHV [≤−2.0], −1.5 YPHV [−1.99 to −1.0], −0.5 YPHV [−0.99 to 0.0], 0.5 YPHV [0.01 to 1.0], 1.5 YPHV [1.01 to 2.0], and 2.5 YPHV [≥2.01]).

### 2.3. Assessment of Physical Fitness Indicators

The experimental protocol consisted of a series of physical performance tests administered in a specific order on the second day of the evaluation. The selected physical fitness assessments were chosen based on their relevance to basketball-specific physical demands and their validation in similar youth populations. The participants first completed three maximum speed tests without a ball, covering distances of 5 m, 10 m, and 20 m, respectively. This was followed by the 20 m dribbling test, which assessed speed with ball control. Finally, participants performed the shuttle run 10 x 5 m and the Illinois test, which evaluated their agility and change of direction abilities. Each participant was given two attempts at each test, with the best result being recorded for subsequent analysis. Performances were evaluated and recorded using a photocell (Witty system, photocells witty gate, Microgate, Bolzano, Italy) and positioned at the starting line and at 5, 10, and 20 m, and were placed 0.7 m above the ground, according to the protocol. On the third day, the participants performed additional tests, including the Sargent test, five-jump test, 1 kg medicine ball throw, and the 20 m shuttle run test, to further assess jump performance, power, and endurance. Prior to the commencement of the testing battery, all participants engaged in a standardized warm-up routine under the supervision of qualified coaches. The warm-up consisted of a low-intensity run followed by a series of dynamic stretching exercises designed to prepare the participants both physically and mentally for the upcoming tests. The dynamic exercises were tailored to improve the flexibility of muscle groups crucial for sprinting, such as the flexors and extensors of the knee, hip, and ankle joints [29].

#### 2.3.1. Sprinting Performances

The sprinting tests (5 m, 10 m, and 20 m) assess speed, a crucial attribute in basketball, particularly for fast breaks and defensive positioning [25]. Maximal sprint speed was evaluated with a 20 m sprint test. The players were instructed to run as fast as possible from a standing position, and both trials were performed with at least 2 min of rest between them. The best time was automatically recorded at 5 m, 10 m, and 20 m (T_5m_, T_10m_, and T_20m_).

#### 2.3.2. The Shuttle Run 10 × 5 m Test

The test was utilized to evaluate the speed and agility of the lower limbs. The marker cones or lines are positioned five meters apart. The participant begins with one foot at a marker. Upon receiving the signal from the timer, the participant sprints to the opposite marker, pivots, and returns to the starting point. This sequence is repeated five times consecutively without pause, covering a total distance of 50 m. Both feet must fully cross each marker line. The time taken to complete the 50 m course is recorded. The equipment needed for this test includes a stopwatch, measuring tape, marker cones, and a flat, non-slip surface [30].

#### 2.3.3. The Illinois Agility Test

The Illinois agility test assesses an athlete’s ability to quickly change direction while maintaining speed. Agility and directional change were essential for dribbling and maneuvering on the court [31]. The test is set up with a rectangular course of 10 m in length and 5 m in width and marked by cones. Four cones are placed at the corners, and four more are positioned centrally in a straight line down the middle, spaced 3.3 m apart. The participant begins the test lying face down behind the start line. On the signal “Go”, they rise as quickly as possible and run through a designated course that involves sprinting, making two 90-degree turns, and weaving through the central cones. The time taken to complete the course, from start to finish, is recorded as the agility score [31].

#### 2.3.4. Sargent Test

The Sargent test aims to assess explosive strength in the lower limbs [32]. The athlete positions themselves sideways against a wall with one arm raised, marking the highest point they can reach without lifting their foot from the ground. They then execute a maximal vertical jump using both legs, ensuring full limb movement and lower-limb joint flexibility, aiming to touch the wall at its highest accessible point. The test quantifies the distance between these two points. Each athlete performs three jumps with a minimum 45 s interval between attempts, with the best recorded for analysis.

#### 2.3.5. Five Jump Test

The test is currently utilized in field settings to assess the explosive power of athletes’ lower limbs and to measure the distance covered. The test was conducted indoors in a hall. Each player begins the test with feet together and chooses which foot to step forward first. Throughout the last stride, the players are instructed to land with their feet together. Before completing the tests, each player underwent several familiarization trials. Performance was expressed relative to leg length (5JT relative) [33]. Jumping tests, including the vertical jump and the horizontal jump five jump test (5JT), capture explosive power relevant for rebounding and shot-blocking [34].

#### 2.3.6. Medicine Ball Throwing Test

To assess the muscular strength of the upper limbs, the medicine ball-throwing test was conducted following a specific protocol [35].

A 1 kg medicine ball, a bench, and a measuring tape were utilized. Three testers were involved. One monitored the technique of each trial, while the other two observed where the medicine ball landed, ensuring consensus between them. Before commencing the test, the bench was positioned upright, and the measuring tape was securely placed on the floor to accurately measure the distance of each throw. The participants were instructed to sit upright on the bench with their backs supported and feet flat on the floor. In terms of the throwing technique, the participants were instructed to hold the medicine ball at chest level. To complete the testing protocol, the participants were instructed to forcefully extend their elbows before releasing the medicine ball. Throughout the testing session, the participants were reminded to maintain contact between their back and the bench and to keep their feet on the floor; any loss of contact required a retry of the trial. Each participant performed three trials with a 90 s rest period between each trial.

#### 2.3.7. The 20 m Shuttle Run Test

The test measures maximal aerobic capacity (VO2 max). The players performed repeated runs over 20-m distances, back and forth between the start line and the finish line marked by cones, at progressively increasing speeds determined by audio signals emitted from a CD player. Between each round, players had a 10 s break, during which they walked around a cone placed 5 m from the start line. If the player is unable to complete two consecutive rounds within the specified time, this will cause the test to be terminated. The assessment of aerobic capacity is crucial for sustaining high-intensity play [36].

### 2.4. Statistical Analyses

Descriptive statistics, including mean and standard deviation (SD), were calculated for each variable. Normal distribution of the data was assessed using the Kolmogorov–Smirnov test. To analyze differences between age and maturity groups, we conducted a one-way ANOVA, followed by Bonferroni post hoc comparisons. Statistical significance was determined with a threshold of (*p* < 0.05). The effect size (ES) was calculated according to Cohen’s method [37], recognizing that relying solely on the *p*-value does not provide insight into the magnitude, direction, or practical relevance of the observed effect [38]. The ES values were interpreted as follows: trivial (<0.35), small (0.35 to 0.8), moderate (0.8 to 1.5), and large (≥1.5).

A multiple linear regression analysis was employed to identify the most predictive anthropometric variables for the dependent variables (physical performances). All statistical analyses were conducted using the Statistical Package for the Social Sciences (SPSS, version 19.0, SPSS Inc., Chicago, IL, USA).

## 3. Results

The data in Table 1 highlight the anthropometric characteristics and physical performances (mean ± SD (ES)) of young male Palestinian basketball players according to age groups. The most notable results emphasize the critical periods of growth and development, particularly between the U-12 and U-14 age groups, where significant changes in anthropometric characteristics and physical performance were observed. Significant differences in YPHV across age groups highlight the growth periods, with large effect sizes (up to 2.65 in U-14), showing the transition from pre- to post-PHV.

Height shows significant growth between U-12 to U-15, with moderate effect sizes (up to 0.92). This is a key indicator of the physical maturation process. Weight increases steadily, with a small effect size (0.86) between U-13 and U-14, indicating an important phase of growth. Significant growth in sitting height and LLL, particularly between U-12 to U-14, with moderate effect sizes (up to 1.06) reflecting overall body growth and the proportionate development of the trunk and lower limbs. Significant increases in arm span and wingspan between U-13 to U-14, with moderate effect sizes (up to 1.12), suggest enhanced upper body growth during these years.

Concerning physical performances, the sprint performances (T_5m_, T_10m_, and T_20m_) marked improvements in sprint times, especially in the T_20m_ sprint, with a large effect size (1.45) between U-12 and U-13, highlighting improved speed as players mature. Improvements in agility and speed endurance, with moderate effect sizes in the shuttle run and Illinois test, show enhanced physical conditioning as the players progress in age. There were significant gains in strength and power, especially in the 1 kg throwing test, where large effect sizes (up to 2.04) indicate substantial improvements in upper body strength, critical for basketball performance.

The correlations between anthropometric and physical parameters and age are presented in Table 2. Height, sitting height, length of lower limbs, arm span, and wingspan all showed strong positive correlations with age, reflecting considerable growth in these dimensions as players mature. In contrast, BMI, body fat, and percentage of fat exhibited minimal changes with age, indicating stability in body composition. The fat-free mass demonstrated a moderate increase with age, suggesting growth in lean body mass. Physical performance also improved significantly with age, as evidenced by faster sprint times, better agility, and increased endurance. Strength and power measurements, including vertical jump height and throwing distance, showed substantial gains, underscoring enhanced physical capabilities as the players grow older.

Anthropometric and physical performance data (mean ± SD (ES)) of young male Palestinian basketball players according to maturity status are presented in Table 3.

The analysis of anthropometric and physical performance variables by maturity status highlights several important trends and significant differences. As players advance through maturity stages, substantial increases in height, weight, sitting height, length of lower limbs, arm span, and wingspan are observed, indicating growth and development. For instance, height increases from 153.49 cm in the −2.5 YPHV group to 180.62 cm in the 2.5 YPHV group, with a significant effect size (ES = 1.26). Similarly, significant differences are noted for weight, with a notable increase from 52.02 kg in the −2.5 YPHV group to 69.77 kg in the 2.5 YPHV group (ES = 0.18 to 0.44), reflecting a growth trend across categories (*p* < 0.05).

BMI shows relatively stable changes, indicating consistent body composition relative to height across maturity stages. Body fat increases moderately from −1.5 to −0.5 YPHV groups (ES = 0.59), with significant differences observed (*p* < 0.05). Fat-free mass also shows significant growth, particularly between −2.5 and −1.5 YPHV (ES = 0.90), suggesting muscle development (*p* < 0.05).

Physical performance improvements are evident with advancing maturity. The sprint times improve significantly, with T_5m_ decreasing from 1.52 s in the −2.5 YPHV group to 1.39 s in the 2.5 YPHV group (ES = 0.72, *p* < 0.05). Agility, as measured by the 10 × 5 m shuttle run and Illinois test, also shows enhanced performance, with significant differences observed across maturity stages. The 20 m shuttle run test (20 m SRT) improves from −2.5 to 2.5 YPHV (ES = 0.11, *p* < 0.05), and the Sargent jump test increases from 35.28 cm to 50 cm (ES = 0.24, *p* < 0.05). Throwing strength, measured by the 1 kg throwing distance, shows marked gains, increasing from 5.87 m in the −2.5 YPHV group to 12.45 m in the 2.5 YPHV group (ES = 0.61, *p* < 0.05). These findings underscore the significant impact of maturity on physical development and performance capabilities in young male basketball players.

**Figure 1:**
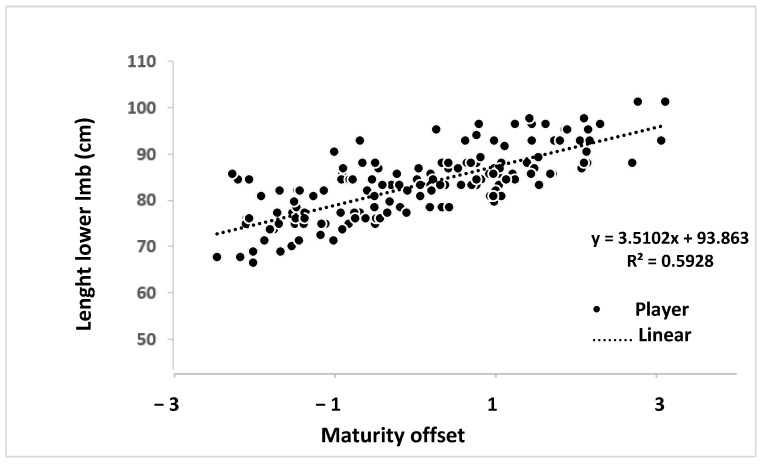
Illustrates the relationship between lower limb length and maturity offset of Palestinian basketball players.

The Figure 1 revealed a strong relationship (R^2^ = 0.593) between LLL and maturity offset among Palestinian basketball players. LLL increased progressively across different maturational stages, with the most significant growth occurring around the timing (PHV).

The full model of multiple physical performance variables, examining the relative contribution of each independent variable to each regression model, is presented in Table 4. The final model of the multiple regression of physical performances in young male Palestinian basketball players is presented in Table 5. The regression analysis identified the following key determinants for physical performance in young male Palestinian basketball players. YPHV is a significant determinant for sprint times (T_5m_, T_10m_, and T_20m_), T_20m_ dribbling, and the 10 × 5 m shuttle run and Illinois test.

Performance on the 20 m SRT is impacted by maturity and sitting height. The Sargent test is impacted by maturity and body fat. LLL is the determinant of the 5JT. The 5JT, relatively, was predicted by LLL and body fat. Finally, the 1 kg throwing performance was predicted by maturity, height, and sitting height.

## 4. Discussion

The study aimed to investigate the anthropometric and physical performance characteristics of male youth basketball players aged 12 to 16 in Palestine, analyze their developmental patterns based on age and maturity, and identify the anthropometric factors affecting physical performance in this population.

Our findings show that anthropometric measurements demonstrated early development, leading to significant improvements in jumping performances and sprinting. In addition, the development of body composition and fat mass seem to influence various physical performances, especially in muscular strength and sprint tests. A noticeable improvement in intermittent endurance performance occurred around the timing of peak height PHV.

Statistical analyses, including full-model and multiple-regression analyses, were performed to determine which anthropometric parameters significantly influenced the performance variables.

Significant differences in anthropometric variables between the age and maturity groups were also shown. In contrast, no significant differences appeared in BMI and body fat percentage. Morphological changes, particularly in the speed of peak height growth, were most evident between ages 13 and 15. Indeed, the onset of the adolescent growth spurt coincides with puberty, during which thyroid hormone, insulin, corticosteroids, and leptin regulate body composition and growth by influencing skeletal mineralization [39], which is further affected by growth hormone mediated by growth hormone-releasing hormone (GHRH) and somatostatin [40].

The absence of significant differences in BMI and body fat percentage in our study is consistent with the findings of [41], who conducted a similar study on Finnish players. The lack of variation in body fat percentage across maturity groups can be attributed to the simultaneous increase in testosterone levels during puberty, which reduces fat mass by inhibiting triglyceride synthesis [42].

We suggest that the timing of somatic growth, particularly PHV, plays also a crucial role, since Philippaerts, et al. [43] found that European male athletes reached PHV at an average age of (13.8 ± 0.8 years). This seems like the average PHV age (13.95 ± 0.5 years) in our Palestinian basketball players but not what was found in Tunisian athletes (14.69 ± 0.69) [10].

In relation to the development of anthropometric characteristics and maturity status, we observed significant changes in key variables as the players progressed through different stages of maturation. Notably, changes were seen in weight, height, and fat-free mass, which is consistent with the findings of [43], where morphological changes during PHV were noted between the ages of 13 and 14. In a previous study, we showed that early-maturing players were generally taller and heavier than their late-maturing counterparts. This aligns also with other studies [24,44], where it was shown that biological maturity affected anthropometric and physical differences.

According to chronological age, Palestinian players exhibit less height and body mass compared to U-13 Polish basketball players [22]. The study by Ramos, et al. [45] demonstrated that Portuguese U-14 players were less tall and less heavy compared to U-14 Polish basketball players. This trend suggests a regional difference in growth patterns and physical development among young athletes, possibly influenced by environmental, genetic, and socioeconomic factors, as socioeconomic status significantly impacts somatic growth [46].

Our study results showed statistically significant differences in all physical measurements between early- and late-maturing groups. In fact, no significant differences were observed for the 5 m sprint and five-jump relative tests. Late-maturing players demonstrated superior performance on the speed and agility tests, whereas early-maturing players excelled in strength and endurance assessments, as evidenced by the study conducted by Guimarães, Ramos, Janeira, Baxter-Jones, and Maia [24].

Additionally, some differences were shown for the Illinois agility test and the vertical jump across maturity groups. This is consistent with studies highlighting the role of increased muscle mass and body size in enhancing athletic performance [47]. This may be due to the fact that early-maturing players experience greater increases in muscle mass, height, and strength during adolescence, providing them with better physical capabilities in basketball-specific tasks.

Our analysis reveals that older age groups tend to excel in several physical performance variables, including speed, agility, jumping ability, and strength. This can be attributed to increased muscle mass, improved neuromuscular coordination, and refined motor skills that develop with age. Conversely, younger players have shown superior performance in aerobic capacity, as measured by the 20 m shuttle run test. This may reflect their higher relative energy efficiency during sustained activities or a greater capacity for cardiovascular endurance at this stage of development. This dichotomy suggests that while maturity confers advantages in certain physical attributes, it may also present challenges in others. This trend aligns with [47], which suggested that players with better physical performances tend to have greater height, muscle mass, and chronological age as the primary indicators of basketball performance. The results also suggest that Palestinian coaches may underestimate the importance of cardiovascular fitness in youth training, which is crucial for enhancing overall physical performance [48], while in contrast, understanding these performance trends can inform talent identification processes.

Chronological age provides a useful reference for understanding growth and maturity. However, biological development does not follow a strictly linear path [4]. Consequently, youths of the same age often exhibit a wide variation in morphological and physiological characteristics. These variations arise from differences in the pace of development, which leads to changes in physical characteristics and performance both prior to and following PHV [49]. This variability poses significant challenges in sports, where competitions are typically organized by age groups to ensure fairness. Maturity differences, however, can confer substantial advantages in performance and influence talent identification [50,51].

Our results reveal significant differences in leg length growth among successive maturity groups. Performance on the Sargent test and the 5JT improved with both age and maturity status, indicating enhanced explosive power in the lower limbs. Notably, while performance in jumping ability increased significantly with age [52], this improvement does not align uniformly with anthropometric gains, as highlighted in various studies [53].

Performances in sprinting improved with age and maturity, with significant changes observed between the U-13 and U-14 age groups for the T_10m_ and T_20m_. Sprint time differences were most notable across maturity groups, specifically from −2.5 YPHV to 0.5 YPHV. These findings align with previous research of Yagüe and De La Fuente [54], who reported speed improvements in the 12 months following PHV. However, they also noted improvements in speed as early as 16 months prior to PHV.

In the same context, the U-13 and U-15 age groups’ Tunisian basketball players showed high speed performance in sprinting. The results let us suggest that performance enhancements are strongly influenced by body mass and ground contact times, as players tend to spend longer durations in ground contact while sprinting. During the pre-PHV phase, stride frequency typically decreases, while stride length increases with advancing maturation. These performance gains can be attributed to factors such as increased limb length and enhanced relative force production [28]. It is important to emphasize that maturation results in significant increases in lean muscle mass, which facilitates improvements in concentric strength and power expression [3].

Furthermore, given that body composition plays an important role in influencing endurance and power performance during adolescence, we attribute the observed increases in lean mass to improvements in oxygen utilization and muscle strength [4]. However, the simultaneous increase in both fat and muscle mass can elevate energy demands, potentially reducing endurance efficiency during prolonged activities [55]. Early-maturing athletes often exhibit increased confidence stemming from their physical advantages, which in turn, positively influences their performance outcomes [56]. In contrast, late-maturing athletes may face reduced self-esteem or frustration, potentially hindering their endurance and power performance [57,58].

A regression analysis showed that maturity was identified as the most crucial determinant for sprint performances (T_5m_, T_10m_, and T_20m_), dribbling, the 10 × 5 m shuttle run, and the Illinois agility tests. This reflects the strong influence of biological development on speed, agility, and coordination, which improve as athletes mature. Aerobic performance (20 m SRT) was predicted by maturity and negatively influenced by sitting height and percentage of fat. Vertical jump and explosive power using the Sargent test were positively influenced by maturity and negatively impacted by body fat. The LLL was the determinant of the 5JT, as longer legs allow for greater stride length and force application, contributing to superior horizontal jump performance. Concerning the 1 kg throwing performance, this was impacted by maturity, height, and sitting height. Maturity contributes to muscular strength, while greater body and segmental dimensions (height, sitting height) enhance leverage and throwing distance.

### Practical Recommendation

The present study establishes the first age- and maturity-specific morphological and fitness investigation for young Palestinian basketball players aged 12–16 years. These benchmarks are invaluable for quantifying and monitoring athletes’ performance and development over time. The findings emphasize the importance of tailoring training programs to an athlete’s maturity status, particularly during the pre-PHV stage.

Individualized training strategies that consider physical characteristics are essential for optimizing performance and supporting long-term athlete development. These strategies enable coaches to tailor their programs to each player’s unique developmental trajectory, ensuring sustained progress while minimizing the risk of injury.

## 5. Conclusions

This study highlights the critical role of anthropometric development and maturity status in shaping the physical performance of young Palestinian basketball players aged 12–16 years. Specifically, LLL emerged as a key contributor to vertical jump performance, while increases in fat-free mass were associated with improvements in strength and power tasks, such as sprinting and throwing. Conversely, fat mass was found to negatively impact speed and explosive power, emphasizing the importance of optimizing body composition during adolescence. Our findings underscore the necessity of considering maturity status alongside chronological age when assessing physical performance and designing training programs for youth athletes. Early-maturing players demonstrated superior strength and power performances, while late-maturing players exhibited advantages in relative agility and endurance. These developmental trajectories highlight the need for tailored training approaches that address the unique physical and maturational characteristics of each athlete. While this study provides valuable data for understanding the developmental trajectories of young Palestinian basketball players, the findings should be interpreted with caution. Including both male and female participants would provide a broader understanding of sex-specific differences in maturity-related physical performance and enhance the applicability of findings to a wider athletic population. The results are specific to this population and may not be directly generalizable to athletes from other regions or socioeconomic backgrounds. Future research with larger, more diverse samples and longitudinal designs is recommended to validate these findings and further explore the interplay between maturity status and athletic performance.

## Figures and Tables

**Table 1:** Anthropometric characteristics and physical performances (mean ± SD (ES)) of youth male Palestinian basketball players according to age group.

Variables	U-12 (ES 12–13)	U-13 (ES 13–14)	U-14 (ES 14–15)	U-15 (ES 15–16)	U-16
Age (y)	11.86 ± 0.25 (4.19) ¥	12.95 ± 0.21 (3.37) ¥	13.96 ± 0.27 (3.96) ¥	14.91 ± 0.26 (4.24) ¥	15.95 ± 0.23
YPHV	−1.59 ± 0.52 (1.24) ¥	−0.93 ± 0.55 (2) ¥	0.02 ± 0.41 (2.65) ¥	1.09 ± 0.39 (1.24) ¥	1.74 ± 0.66
Weight (kg)	53.46 ± 11.87 (0.18)	55.33 ± 9.33 (0.08)	56 ± 8.02 (0.86) ¥	62.93 ± 8.13 (0.22)	65.27 ± 13.46
Height (cm)	158.79 ± 81 (0.25)	160.57 ± 6.23 (0.64) ¥	164.51 ± 6.04 (0.92) ¥	170.23 ± 6.4 (0.78) ¥	175.63 ± 7.49
Sitting height (cm)	78.69 ± 4.58 (0.33)	80.23 ± 4.83 (1.06) ¥	84.5 ± 3.24 (1.02) ¥	87.67 ± 2.99 (0.49)	89.37 ± 4.01
LLL (cm)	89.39 ± 4.54 (0.13)	90.03 ± 4.98 (0.83) ¥	94.17 ± 4.97 (0.81) ¥	97.97 ± 4.45 (0.21)	98.93 ± 4.56
Arm span (cm)	63.02 ± 3.81 (0.37)	64.33 ± 3.42 (0.53)	66.07 ± 3.08 (1.12) ¥	69.87 ± 3.67 (0.49) ¥	71.78 ± 4.05
Wingspan (cm)	160.13 ± 7.44 (0.35)	162.59 ± 6.62 (0.67) ¥	167.61 ± 8.25 (0.85) ¥	174.03 ± 6.96 (0.66) ¥	178.89 ± 7.98
BMI (kg/m^2^)	21.11 ± 3.92 (0.08)	21.37 ± 2.79 (0.22)	20.73 ± 2.99 (0.37)	21.67 ± 1.98 (0.23)	21.04 ± 3.44
Bodyfat (kg)	7.75 ± 5.17 (0.05)	7.98 ± 4.27 (0.31)	9.44 ± 5.28 (0.07)	9.86 ± 5.98 (0.03)	9.64 ± 7.69
Percentage of fat (%)	13.82 ± 5.52 (0.08)	13.38 ± 5.36 (0.09)	13.92 ± 5.98 (0.00)	13.89 ± 6.02 (0.12)	13.08 ± 7.51
Fat-free mass (kg)	25.87 ± 6.01 (0.25)	27.38 ± 6.01 (0.76) ¥	31.18 ± 3.97 (0.32)	32.45 ± 3.92 (0.59)	30.11 ± 4.02
T_5m_ (s)	1.63 ± 0.32 (0.2)	1.579 ± 0.24 (0.73) ¥	1.43 ± 0.16 (0.48)	1.37 ± 0.11 (0.25)	1.42 ± 0.14
T_10m_ (s)	2.67 ± 0.42 (0.39)	2.52 ± 0.34 (0.88) ¥	2.22 ± 0.36 (0.47)	2.06 ± 0.34 (0.47)	1.91 ± 0.31
T_20m_ (s)	4.25 ± 0.47 (0.1)	4.21 ± 0.34 (1.45) ¥	3.63 ± 0.46 (0.15)	3.57 ± 0.35 (0.44)	3.43 ± 0.28
T_20m_ dribbling (s)	5.18 ± 0.56 (0.56) ¥	4.89 ± 0.46 (1.23)	4.29 ± 0.54 (0.44)	4.06 ± 0.47 (0.36)	3.89 ± 0.49
10 × 5 m shuttle run (s)	19.91 ± 1.73 (0.14)	19.67 ± 1.65 (0.62) ¥	18.67 ± 1.55 (0.36)	18.09 ± 1.68 (0.49)	17.31 ± 1.49
Illinois test (s)	21.34 ± 1.47 (0.29)	20.92 ± 1.44 (0.46)	20.24 ± 1.51 (0.52) ¥	19.34 ± 1.92 (0.61) ¥	18.19 ± 1.83
20 m SRT (mL/kg/min)	38.13 ± 3.15 (0.37)	39.25 ± 2.89 (0.62)	41.17 ± 3.32 (0.17)	41.82 ± 4.29 (1.29) ¥	47.91 ± 5.11
Sargent test (cm)	34.07 ± 5.06 (0.77) ¥	38 ± 5.17 (1.34) ¥	45.53 ± 6.03 (0.23)	47.23 ± 8.75 (0.03)	47.52 ± 8.66
5JT (m)	17.49 ± 0.95 (0.00)	17.49 ± 1.04 (0.86) ¥	18.35 ± 0.96 (0.95) ¥	19.22 ± 0.88 (0.11)	19.32 ± 0.92
5JT Relative	0.22 ± 0.02 (0.25)	0.22 ± 0.01 (0.06)	0.22 ± 0.01 (0.24)	0.22 ± 0.01 (0.46)	0.22 ± 0.01
1 kg throwing (m)	6.17 ± 1.09 (1.07) ¥	7.29 ± 1.02 (1.55) ¥	8.64 ± 0.72 (1.35) ¥	9.86 ± 1.08 (2.04) ¥	12.61 ± 1.62

ES: effect size; YPHV: years from peak height velocity; LLL: length of lower limb; BMI: body mass index.;T_5m_: time 5 m sprint; T_10m_: time 10 m sprint; T_20m_: time 20 m sprint; T_20m_ dribbling with ball; 10 × 5 m shuttle run: 10 repetitions of 5 m shuttle run; 20 m SRT: 20 m shuttle run; 5JT: five jump test; 5JT Relative: five jump test relative; 1 kg throwing: throwing 1 kg medicine. ¥ Significant difference in age category vs. following age category in the anthropometric characteristics and physical performances physical characteristics for male basketball players (*p* < 0.05).

**Table 2:** Correlation coefficients between anthropometric and physical performances and age of male Palestinian basketball players.

Anthropometric Parameters	Correlation Coefficient (r)	*p* Value
Weight (kg)	0.39	0.001
Height (cm)	0.67	0.001
Sitting height (cm)	0.71	0.001
Length of lower limbs (cm)	0.63	0.001
Arm span (cm)	0.67	0.001
Wingspan (cm)	0.68	0.001
BMI (kg/m^2^)	0.01	0.922
Body fat (kg)	0.14	0.090
Percentage of fat (%)	0.02	0.784
Fat-free mass (kg)	0.36	0.001
Physical performances		
T_5m_ (s)	−0.42	0.001
T_10m_ (s)	−0.63	0.001
T_20m_ (s)	−0.63	0.001
T_20m_ dribbling (s)	−0.69	0.001
10 × 5 m shuttle run (s)	−0.51	0.001
Illinois test (s)	−0.56	0.001
20 m SRT (mL/kg/min)	0.61	0.001
Sargent test (cm)	0.58	0.000
5JT (m)	0.62	0.001
5JT Relative	−0.13	0.106
1 kg throwing (m)	0.88	0.001

BMI = body mass index; T_5m_: time 5 m sprint; T_10m_: time 10 m sprint; T_20m_: time 20 m sprint; T_20m_ dribbling with ball; 10 × 5 m shuttle run: 10 receptions of 5 m shuttle run; 20 m SRT: 20 m shuttle run; test 5JT: five jump test; 5JT Relative: five jump test relative; 1 kg throwing: throwing 1 kg medicine.

**Table 3:** Descriptive statistics (mean ± SD (ES)) of anthropometric parameters and physical performances according to maturity status of youth male Palestinian basketball players according to maturity status.

Variables	−2.5 ES (−2.5 vs. −1.5)	−1.5 ES (−1.5 vs. −0.5)	−0.5 ES (−0.5 vs. 0.5)	0.5 ES (0.5 vs. 1.5)	1.5 ES (1.5 vs. 2.5)	2.5
	(n = 10)	(n = 28)	(n = 33)	(n = 37)	(n = 29)	(n = 13)
YPHV (years)	−2.15 ± 0.14 (3.54) ¥	−1.46 ± 0.25 (3.67) ¥	−0.54 ± 0.25 (3.79) ¥	0.54 ± 0.32 (2.81) ¥	1.37 ± 0.28 (2.99) ¥	2.37 ± 0.39
Weight (kg)	52.02 ± 10.83 (0.18)	50.33 ± 8.36 (0.89) ¥	58.44 ± 9.86 (0.01)	58.38 ± 7.68 (0.62) ¥	64.31 ± 11.49 (0.44)	69.77 ± 13.62
Height (cm)	153.49 ± 6.87 (0.65) ¥	157.75 ± 6.43 (0.94) ¥	162.97 ± 4.82 (0.85) ¥	167.11 ± 4.95 (1.17) ¥	173.48 ± 5.99 (1.26) ¥	180.62 ± 5.35
Sitting height (cm)	73.47 ± 2.95 (1.56) ¥	78 ± 2.79 (1.86) ¥	82.97 ± 2.56 (1.35) ¥	86.14 ± 2.12 (0.85) ¥	88.17 ± 2.66 (2.28) ¥	93.31 ± 1.84
LLL (cm)	87.28 ± 6.29 (0.23)	88.46 ± 3.89 (0.86) ¥	91.73 ± 3.67 (1.12) ¥	95.92 ± 3.68 (0.67) ¥	98.59 ± 4.11 (0.91) ¥	102.31 ± 4.11
Arm span (cm)	60.48 ± 3.81 (0.66) ¥	62.82 ± 3.17 (0.93) ¥	65.39 ± 2.33 (0.77) ¥	67.49 ± 3.09 (1.12) ¥	71.07 ± 3.31 (1.33) ¥	74.77 ± 2.28
Wingspan (cm)	155.19 ± 67 (0.65)	159.48 ± 6.29 (0.98) ¥	164.79 ± 4.47 (1) ¥	170.51 ± 6.95 (1)	177.14 ± 6.63 (1.27) ¥	184.54 ± 5.36
BMI (kg/m^2^)	22.07 ± 4.51 (0.52)	20.17 ± 2.68 (0.58) ¥	21.94 ± 3.06 (0.35)	20.92 ± 2.67 (0.13)	21.28 ± 3.62 (0.01)	21.27 ± 3.04
Body fat (kg)	6.84 ± 3.63 (0.06)	6.62 ± 3.57 (0.59)	9.25 ± 5.33 (0.02)	9.38 ± 5.89 (0.09)	10.02 ± 6.89 (0.14)	11.04 ± 7.82
Percentage of fat (%)	13.38 ± 3.87 (0.01)	13.33 ± 5.67 (0.14)	14.12 ± 5.63 (0.19)	13.04 ± 5.98 (0.11)	13.75 ± 7.42 (0.09)	14.48 ± 7.63
Fat-free mass (kg)	24.78 ± 6.92 (0.03)	25 ± 5.66 (0.90) ¥	29.78 ± 5.04 (0.35)	31.29 ± 3.39 (0.19)	31.35 ± 4.57 (0.06)	31.61 ± 3.86
T_5m_ (s)	1.52 ± 0.17 (0.72) ¥	1.67 ± 0.35 (0.84) ¥	1.48 ± 0.18 (0.46)	1.41 ± 0.15 (0.15)	1.39 ± 0.12 (0.12)	1.39 ± 0.17
T_10m_ (s)	2.57 ± 0.22 (0.32)	2.69 ± 0.49(0.74) ¥	2.38 ± 0.35 (0.72) ¥	2.12 ± 0.37 (0.27)	2.02 ± 0.32 (0.31)	1.92 ± 0.31
T_20m_ (s)	4.19 ± 0.38 (0.32)	4.32 ± 0.48 (0.81) ¥	3.94 ± 0.46 (0.66) ¥	3.65 ± 0.42 (0.52)	3.47 ± 0.28 (0.11)	3.43 ± 0.35
T_20m_ dribbling (s)	5.07 ± 0.37 (0.15)	5.14 ± 0.62 (0.82) ¥	4.66 ± 0.55 (0.91) ¥	4.16 ± 0.55 (0.39)	3.97 ± 0.45 (0.01)	3.97 ± 0.52
10 × 5 m shuttle run (s)	19.69 ± 1.69 (0.14)	19.93 ± 1.67 (0.32)	19.39 ± 1.71 (0.49) ¥	18.54 ± 1.73 (0.78) ¥	17.34 ± 1.36 (0.04)	17.39 ± 1.48
Illinois test (s)	20.95 ± 1.32 (0.29)	21.36 ± 1.54 (0.42)	20.74 ± 1.41 (0.41)	20.07 ± 1.84 (1.09) ¥	18.23 ± 1.54 (0.05)	18.31 ± 1.99
20 m SRT (mL/kg/min)	39.13 ± 2.20 (0.35)	38.09 ± 3.66 (0.37)	39.23 ± 2.48 (1.25) ¥	43.25 ± 3.95 (0.34)	44.92 ± 5.82 (0.11)	45.57 ± 6.69
Sargent test (cm)	35.28 ± 6.96 (0.01)	35.36 ± 4.98 (0.79) ¥	40.03 ± 6.82 (0.72) ¥	45 ± 6.98 (0.36)	47.97 ± 9.34 (0.24)	50 ± 7.33
5JT (m)	16.99 ± 1.19 (0.39)	17.39 ± 0.93 (0.39)	17.75 ± 0.78 (1.32) ¥	18.75 ± 0.871 (0.73) ¥	19.32 ± 0.84 (0.72) ¥	19.95 ± 0.89
5JT Relative	0.23 ± 0.02 (0.39)	0.22 ± 0.01 (0.77) ¥	0.21 ± 0.01 (0.45)	0.22 ± 0.01 (0.16)	0.22 ± 0.01 (0.87)	0.21 ± 0.01
1 kg throwing (m)	5.87 ± 0.89 (0.96)	6.77 ± 0.97 (0.68) ¥	7.56 ± 1.38 (1.34) ¥	9.43 ± 1.42 (1.12) ¥	11.32 ± 1.97 (0.61) ¥	12.45 ± 1.74

ES: effect Size; YPHV: years from peak height velocity; LLL: =length of lower limb; BMI: body mass index.; T_5m_: time 5 m sprint; T_10m_: time 10 m sprint; T_20m_: time 20 m sprint; T_20m_ dribbling with ball; 10 × 5 m shuttle run: 10 repetitions of 5 m shuttle run; 20 m SRT: 20 m shuttle run; 5JT: five jump test; 5JT Relative: five jump test relative 1 kg throwing: throwing 1 kg medicine. ¥ Significant difference in age category vs. following age category in the anthropometric characteristics and physical performance characteristics for male basketball players (*p* < 0.05).

**Table 4:** Full model predicting physical performance variables according to maturity and anthropometric variables.

Variables	Constant	YPHV	Weight (kg)	Height (cm)	Sitting Height (cm)	LLL (cm)	Arm Span (cm)	Wingspan (cm)	BMI (kg/m^2^)	Body Fat (kg)	Percentage of Fat (%)	Fat-Free Mass (kg)	R-Deux	See
T_5m_ (s)	0.228	−0.518	1.202	−0.005	−0.211	0.409	−0.234	−1.581	1.256	0.045	−0.083	−0.178	0.228	0.21145
T_10m_ (s)	−0.116	−0.827	−0.062	0.247	0.142	−0.206	0.240	−0.084	0.066	0.023	−0.087	−0.070	0.365	0.37374
T_20m_ (s)	1.055	−0.812	0.382	0.123	−0.136	0.540	−0.506	−0.314	0.216	0.029	−0.041	−0.006	0.421	0.40832
T_20m_ dribbling (s)	6.701	−0.970	−0.234	0.274	0.012	0.392	−0.462	0.656	−0.565	0.126	−0.134	−0.054	0.480	0.52366
10 × 5 m shuttle run (s)	7.809	−0.671	−0.128	0.213	0.135	0.387	−0.098	−0.593	0.360	0.210	−0.185	−0.014	0.285	1.64812
Illinois test (s)	11.473	−0.686	−0.311	0.314	0.189	0.195	0.022	−0.574	0.338	0.260	−0.201	0.010	0.361	1.64535
20 m SRT (mL/kg/min)	13.374	1.029	1.147	−0.471	−0.076	−0.339	−0.115	−1.066	0.859	−0.041	−0.112	−0.099	0.402	4.09298
Sargent test (cm)	114.624	0.713	−0.476	−0.193	−0.091	0.431	−0.113	0.356	−0.418	−0.124	0.116	0.268	0.417	6.94364
5JT (m)	12.661	0.141	−0.544	−0.114	0.997	0.013	0.026	0.726	−0.537	−0.064	−0.018	0.024	0.882	0.44038
5JT Relative	0.364	0.192	−0.676	−1.493	1.325	0.029	0.057	0.881	−0.684	−0.127	0.010	−0.005	0.739	0.00607
1 kg throwing (m)	29.999	1.304	0.283	−0.762	−0.082	−0.135	0.053	0.223	−0.151	0.026	−0.075	−0.019	0.750	1.30034

YPHV: years from peak height velocity; LLL: length of lower limb; BMI = body mass index; T_5m_: time 5 m sprint; T_10m_: time 10 m sprint; T_20m_: time 20 m sprint; T_20m_ dribbling with ball; 10 × 5 m shuttle run: 10 receptions of 5 m shuttle run; 20 m SRT: 20 m shuttle run; test 5JT: five jump test; 5JT relative: five jump test relative; 1 kg throwing: throwing 1 kg medicine; See: standard error of estimation.

**Table 5:** Regression analysis of physical performance (dependent variables) and anthropometric variables (independent variables) (*p* < 0.05).

Variables	(Constant)	YPHV	Weight (kg)	Height (cm)	Sitting Height (cm)	LLL (cm)	BMI (kg/m^2^)	Body Fat (kg)	Percentage of Fat (%)	Fat-Free Mass (kg)	R-Deux
T_5m_ (s)	1.486	−0.389	-	-	-	-	-	-	-	-	0.151
T_10m_ (s)	2.288	−0.582	-	-	-	-	-	-	-	-	0.338
T_20m_ (s)	3.835	−0.622	-	-	-	-	-	-	-	-	0.387
T_20m_ dribbling (s)	4.485	−0.656	-	-	-	-	-	-	-	-	0.430
10 × 5 m shuttle run (s)	18.779	−0.498	-	-	-	-	-	-	-	-	0.248
Illinois test (s)	20.063	−0.557	-	-	-	-	-	-	-	-	0.310
20 m SRT (mL/kg/min)	75.832	0.948	-		−0.432	-	-	-	−0.174		0.369
Sargent test (cm)		0.581	−0.182	-	-	-	-	-	-	0.233	0.390
5JT (m)	0.606		-	-	-	0.932	-	-	-	-	0.869
5JT Relative	0.259	0.223	-	-	-	1.272	-	−0.109	-	-	0.729
1 kg throwing (m)	25.269	1.299	-	0.258	−0.762	-	-	-	-	-	0.745

YPHV: years from peak height velocity; LLL: length of lower limb; BMI = body mass index; T_5m_: time 5 m sprint; T_10m_: time 10 m sprint; T_20m_: time 20 m sprint; T_20m_ dribbling with ball; 10 × 5 m shuttle run: 10 receptions of 5 m shuttle run; 20 m SRT: 20 m shuttle run; test 5JT: five jump test; 5JT Relative: five jump test relative; 1 kg throwing: throwing 1 kg medicine; See: standard error of estimation.

## Data Availability

The data that support the findings of this study are available from the corresponding author upon reasonable request.
